# Lessons for public health campaigns from analysing commercial food marketing success factors: a case study

**DOI:** 10.1186/1471-2458-12-139

**Published:** 2012-02-21

**Authors:** Jessica Aschemann-Witzel, Federico JA Perez-Cueto, Barbara Niedzwiedzka, Wim Verbeke, Tino Bech-Larsen

**Affiliations:** 1 MAPP-centre, Aarhus School of Business, Aarhus University, Haslegaardsvej 10, 8210 Aarhus, Denmark; 2 Department of Agricultural Economics, Ghent University, Coupure links 653, B-9000 Ghent, Belgium; 3 Institute of Public Health, Jagiellonian University Medical College, Krakow, ul. Św. Anny 12, 31-008 Kraków, Poland

## Abstract

**Background:**

Commercial food marketing has considerably shaped consumer food choice behaviour. Meanwhile, public health campaigns for healthier eating have had limited impact to date. Social marketing suggests that successful commercial food marketing campaigns can provide useful lessons for public sector activities. The aim of the present study was to empirically identify food marketing success factors that, using the social marketing approach, could help improve public health campaigns to promote healthy eating.

**Methods:**

In this case-study analysis, 27 recent and successful commercial food and beverage marketing cases were purposively sampled from different European countries. The cases involved different consumer target groups, product categories, company sizes and marketing techniques. The analysis focused on cases of relatively healthy food types, and nutrition and health-related aspects in the communication related to the food. Visual as well as written material was gathered, complemented by semi-structured interviews with 12 food market trend experts and 19 representatives of food companies and advertising agencies. Success factors were identified by a group of experts who reached consensus through discussion structured by a card sorting method.

**Results:**

Six clusters of success factors emerged from the analysis and were labelled as "data and knowledge", "emotions", "endorsement", "media", "community" and "why and how". Each cluster subsumes two or three success factors and is illustrated by examples. In total, 16 factors were identified. It is argued that the factors "nutritional evidence", "trend awareness", "vertical endorsement", "simple naturalness" and "common values" are of particular importance in the communication of health with regard to food.

**Conclusions:**

The present study identified critical factors for the success of commercial food marketing campaigns related to the issue of nutrition and health, which are possibly transferable to the public health sector. Whether or not a particular factor contributes to future success depends on the specific context of use, the combination of factors and the environment. Consideration of the specific applicability of the success factors identified in this study during the design of marketing activities could benefit public sector food and health-related campaigns.

## Background

Who would have thought 10 years ago that consumers, as part of their daily routine, would open a tiny bottle of drinkable yoghurt to strengthen their bodily defences? Today, despite consumers reportedly doubting claims that sound too much like a marketing fad [1], this type of product is found in many European households [2]. Commercial food and beverage marketing is clearly influential, although the high failure rate of new food product launches indicates that there are also limits to its power [3]. Despite food marketing's likely negative role in the obesity pandemic [4-7], as well as the cultural divide between the public health and the marketing sector [8-10], some particularly successful commercial campaigns might still lead public policymakers to question what the recipe for success is.

It is not only curiosity, but the need for novel approaches that induces policymakers to consider adopting new approaches based on social marketing techniques [9,11-13]. Obesity is a severe threat to public health. In response to the World Health Organisation's Global Strategy for Diet, Physical Activity and Health [14] numerous national food and nutrition policies have been developed to promote healthy eating. However, many of these policies have merely translated into educational communication campaigns, whose impact on actual behaviour tends to be limited [15-17], largely short-term [18], and not effective enough [19]. There is also a lack of effective measurement of these campaigns' intervention activities [20]. Social marketing, as a relatively new but promising approach toward public policy activities, requires further investigation and refinement in Europe.

Social marketing was defined as early as the 1970s as applying marketing in "programs calculated to influence the acceptability of social ideas" [21]. Social marketing describes efforts that use marketing techniques in a non-commercial setting in an adapted way. It is also described as efforts to influence the "voluntary behaviour of target audiences to improve their personal welfare and that of their society" [22].

Adopting social marketing principles in public health campaigns is relevant since these campaigns advocate healthy eating as a topical social idea, they target food choice, which is a clear example of voluntary behaviour, and influencing food choice can potentially have a huge impact on personal and societal welfare. Efforts to improve healthy eating have already been targeted with the help of marketing techniques in a few, but steadily growing number, of non-commercial campaigns [4,13,23,24]. Despite recognised differences between health communication and social marketing, the significance of social marketing as a valuable approach to public health communication has been acknowledged [25]. Rayner [26] called it "a frame of reference or a mindset for helping examine, understand and provide insight into issues and enhance impact and effectiveness' of interventions aimed at behaviour change".

The European Union (EU) funded research project EATWELL (Interventions to Promote Healthy Eating Habits: Evaluation and Recommendations) aims at analysing the efficacy of previous public healthy eating interventions and identifying promising developments for the future [27]. This work includes a review of the types and reported effectiveness of public sector healthy eating interventions and campaigns [20,28,29] and a reanalysis of earlier data sets to explore the extent of impact of the public efforts [e.g. [30]]. This paper reports on the next phase of the EATWELL project, namely to gather ideas for future improvement of public health efforts by looking at learnings from sectors other than the public health domain. The aim is to assess the learnings from a series of successful commercial and health-related food and beverage marketing campaigns. Apart from conducting an empirical study, the unique approach of the work reported in this paper consists, first, of selecting only food cases deemed commercially successful, and, second, of focusing on cases where nutrition and health, or health-related well-being, plays a role in either the product characteristics or the communication strategy. The objective of this paper is to present the key success factors identified in an empirical case study, so as to enable their use in future social marketing campaigns for public health nutrition purposes.

## Methods

The present study builds on the concept of key success factors. Key success factors (KSF) enable a company to differentiate itself from its competitors and to offer a distinct value to customers or consumers [31]. The unique selling proposition or USP [32] chosen for communication is linked to a KSF. KSF stem from different sources, such as the macro-environment, the sector in question (such as the food industry), the competitive strategy, or the temporal factors of influence within the company [33]. This implies a distinction between the external factors one cannot influence, but can only monitor and eventually act upon, and the internal factors that can be built on or developed further. It also implies that KSF can be looked at from different levels, such as the macro and micro level. Furthermore, success factors are interrelated as well as "embedded in a network of other relevant skills and resources" [33]. Apart from research into KSF in areas such as mature markets and industrial products [34], new product development [35] and food markets [3,36], detecting success factors is also important to evaluate communication and advertising effectiveness. The latter information is collected and reported by advertising award institutions such as the European Association of Communications Agencies (EACA) and the Institute of Practitioners in Advertising (IPA) in the United Kingdom [37].

A case study approach was used to explore success factors in recent commercial marketing activities. Case studies, often used in social sciences, have the advantage of allowing the researcher to gather and generate knowledge and experiences about complex phenomena that are closely connected to a particular context [38,39]. In business education they are valued because "by looking at many cases, one develops an understanding, an intuition for business ecology, which, in future problem-solving situations, will improve the ability to look at the right cues in the environment and come up with the right strategic decision" [33]. Merriam [38] recommends the case study methodology for "studying educational innovations, evaluating programs, and informing policy". It is thus an appropriate approach for identifying insights for future healthy eating policies and gathering insightful, practically relevant examples.

To ensure the quality of the research process with regard to validity, reliability and reflexivity, similar procedures are suggested for the conducting of case studies as for qualitative exploratory research in general [38-43]. Therefore, a harmonised study protocol providing objectives and procedures was developed prior to the purposive sampling of cases. The research process consisted of several steps to allow for critical reflection. Multiple cases, sources and types of materials were used, multiple researchers were involved, and the process allowed for feedback from key informants.

**Figure 1:**
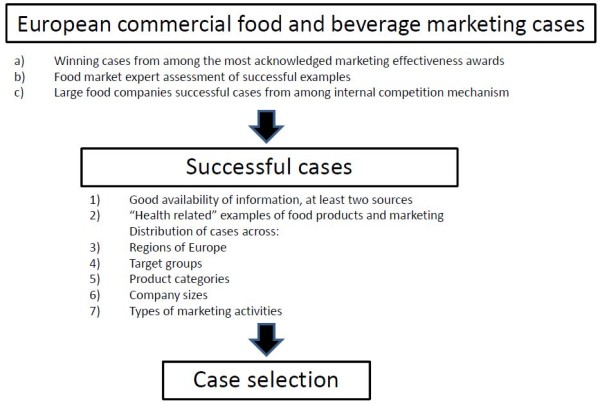
**Selection process for marketing cases**.

The selection of suitable cases is crucial for a successful case study. The European commercial food market offers a vast number of food and beverage marketing campaigns, but only those that were actually successful in changing consumer behaviour are suitable for deducing valuable teachings. Therefore, this was the first criterion for campaign selection. However, general market data rarely allows individual campaign's effects to be singled out, and information about the success criteria for campaigns is largely proprietary data. To overcome these challenges, three methods of identifying suitable food and beverage marketing cases from the past 5 years were chosen (see Figure 1 for an illustration of the selection process):

a) Cases were selected from those acknowledged by marketing effectiveness awards, on the grounds that the award reports contain actual market data as proof of campaign effectiveness, and that these cases have been assessed by an expert committee, which acts as a proxy for a peer-review process.

b) Twelve food market experts (see Table 1), through interviews and email correspondence, identified examples of successful campaigns. This method was chosen because these individuals have knowledge of campaigns that are either too new to have been recognised by awards, or that were not eligible for the awards, because of being based on alternative marketing approaches rather than traditional advertising techniques.

**Table 1:** List of the experts interviewed in the search for cases

**Nr**.	Source/Reason	Contact	Name, affiliation
1.	Acknowledged food market expert	Phone	Em. Professor of Food Marketing, UK

2.	Member of World Federation of Advertisers (WFA), which is a member of the platform on diet, physical activity and health	Phone	ISBA UK Incorporated Society of British Advertisers, UK

3.	Recommendation by other expert	Phone	Consultant, Sweden

4.	One of the large food manufacturers in the EU	Phone	Danone, France

5.	Member of platform on diet, physical activity and health, recommendation by other expert	Phone	WFA Brussels, World Federation of Advertisers

6.	Recommendation by other expert	Phone	Business 4 life, UK

7.	Member of platform on diet, physical activity and health	Phone	European Heart Network (EHN)

8.	Functional food market expert, author	E-Mail	Institute for Marketing & Innovation, University Wien

9.	Acknowledged food market expert	E-Mail	Bioplus AG, Switzerland

10.	Involvement in reformulation activities in the UK, recommendation by other expert	E-Mail	Food and Drink Federation UK

11.	Functional food market expert, author	E-Mail	Hero Spain

12.	Food researcher in the UK	E-Mail	Department of Psychology, University of Surrey

c) Large food companies were contacted and asked to select and provide successful campaigns from their internal competition process. This method allowed the authors to access cases that were not eligible for awards due to company policy, and material that was not accessible elsewhere. It must be emphasised that the companies did not, however, have an influence on the authors's final choice of which cases to include.

Documents selected for use in the case study were mainly drawn from the award documents of the EACA Euro Effie awards, the national Polish Effie awards, the UK's IPA effectiveness awards, the Spanish Eficacia awards and the European IMC awards for Integrated Marketing Communications. Apart from the award documents, several expert reports were received from the consultant New Nutrition Business and internal documents on possible cases were provided by the companies or agencies contacted. Seven telephonic interviews were conducted with food market trend experts, and a further five experts gave valuable advice via email (see Table 1). Keywords related to potentially interesting brands or campaigns were entered into Internet search engines to access additional publicly accessible documents and online media reports, and into YouTube for an indication of the popularity, content and format of TV advertisements.

Out of the 60 commercially successful cases identified, 27 were selected for further analysis using purposive sampling. Ten cases had won awards, 12 were recommended by the experts, and six were suggested by large food companies (three examples from Nestlé, two from Kraft foods and one from Unilever). Selection criteria included information being available from at least two different sources, and a distribution of cases across different regions of Europe, target groups such as age group, product categories, company size and type of marketing activities. Marketing activities encompassing different types of marketing cases, such as new product launches, product reformulations, new communication measures and cause marketing were selected, as well as examples of generic marketing and retailer activities for retail brands and assortments. Furthermore, selection focused on nutrition-related examples of food products and marketing in the broad sense, meaning that products either had to have favourable nutritional characteristics when compared with other foods of the same category (such as ice cream that had been reformulated to be healthier), or the campaign had to communicate health-related aspects as possibly perceived by consumers (such as the promotion of a passion for outdoor sports in relation to a chocolate bar). It is important to note that the investigators did not attempt to judge which foods are healthy, or whether a specific nutrition and health-related aspect in a campaign was actually promoting health. The aim was rather to identify successful marketing activities and communication approaches so as to extract potential success factors which, when transferred to public health promotion activities, could potentially improve their success and effectiveness.

During the data gathering stage, additional documents were collected and19 personal interviews were conducted - 16 with representatives of food companies and three with representatives of the advertising agencies for the selected products and campaigns. A semi-structured interview guide was used (see Table 2) and the interviews were audio-recorded and transcribed verbatim for analysis.

**Table 2:** Semi-structured interview guide

**Nr**.	Aim	Main question
1.	Talking about the brand/product	• We are filling out a table with information for every case, and there are some blank spaces here. I would like to ask you: [insert specific questions if needed] • What are the most important distinguishing characteristics of the product, the brand and the company, as compared with the main competitors?
2.	Talking about the specific campaign in focus	• Please recount the story of how the campaign developed and "did the trick" of influencing consumer behaviour successfully. Please start from the initial situation and problem, the idea generation, the development of the campaign, what it consisted of, and how you measured the successful outcome. [Use of probing questions if needed]
3.	The relation to the project "EATWELL"	• How would you describe the picture/concept of food, diet and health that the product and the related campaign are painting? • Maybe you have some consumer research data or at least some hints (or guesses) as to how diets and eating habits changed in relation to your product and campaign. Can you tell me a bit more about this? • If public policy makers would use this campaign as a blue-print for a campaign for healthier eating today - what should be kept, and what should be changed?

The probing questions are not included

**Figure 2:**
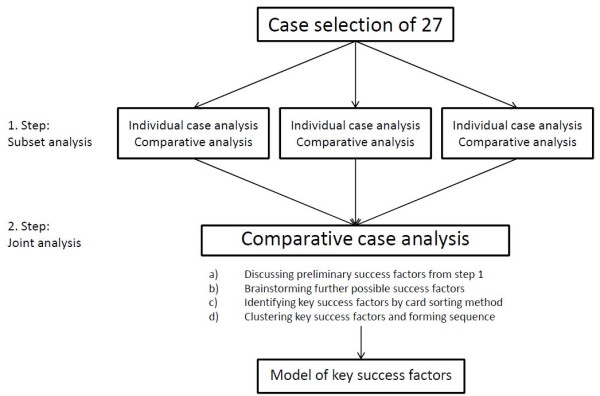
**Analysis process in the case study analysis**.

**Figure 3:**
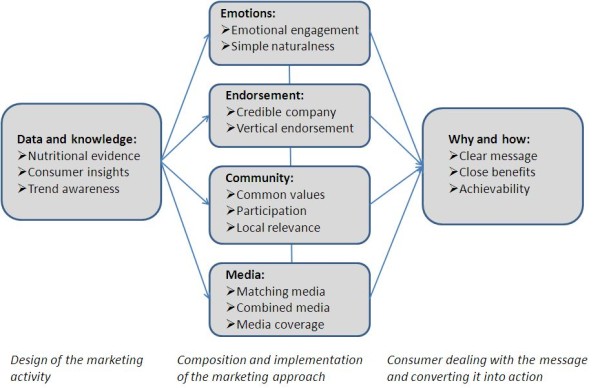
**Model of key success factors in commercial food marketing based on case study results**.

The data analysis process is depicted in Figure 2. The first step in analysing the data collected from the 27 cases was to assign a third of the cases to each project partner to conduct a case-by-case analysis and a comparative analysis of the subset. To control for reflexivity bias, at least two researchers read and reviewed the material in-depth and summarised the content of each case into a concise description based on a uniform template that factually described the case and noted observations or comments on the case. Possible success factors were preliminarily listed within the template. The second step was a joint comparative case analysis where each project partner prepared a presentation of their cases as well as a draft comparative analysis of their set of cases. These materials were shared during a 2-day workshop where authors and project partners with backgrounds in public health nutrition, consumer science, food marketing and advertising participated (see acknowledgments). The comparative analysis followed a stepwise procedure. Initially, individual cases, the preliminary list of success factors, and the three comparative analyses of the subsets were discussed by the group. Thereafter, additional potential success factors were brainstormed, using the information gathered in the first step as the point of departure. These success factors were written on paper cards and displayed on a board for better visualization. Next, the number of possible success factors was reduced using a card sorting method to identify the KSF: The discussion was facilitated by dividing the cards with similar meaning into piles. Each pile of success factors was elicited from several of the cases. Finally, the KSF were clustered into higher-order categories by combining piles that were closely related and deciding on a name that describes what they have in common. Clusters were formed because, although factors were different enough in type and application to be regarded as independent constructs, several appeared closely related enough to allow them to be grouped in a meaningful higher-order category. The clusters were arranged hierarchically and presented in a model that reflects the sequence in which they affect a marketing activity (see Figure 3). The card sorting method is adapted from discussion facilitation techniques and is similar to the multiple sorting technique [44].

## Results

### Relational model of success factors

Six clusters of KSF emerged from the analysis. Figure 3 presents the model of key success factors and the higher-order categories agreed upon in the analysis. It illustrates that the clusters are important in (from left to the right) the design of the marketing activity (the 'homework' behind a successful market launch); the actual composition and implementation of the marketing approach; and the consumer processing the message and converting it into action. Each cluster was given an inclusive title agreed upon during the expert workshop. A better understanding can be gained through the following description of the KSF within each cluster and the provision of easy to understand contextual examples. The KSF identified as most important for the success of each example mentioned in the text are presented in Table 3.

**Table 3:** Commercial food marketing cases and the most important success factors

Cases	Clusters and key success factors					
**Company/brand, product, case/campaign, country**	**Data and knowledge**	**Emotions**	**Endorsement**	**Community**	**Media**	**Why and how**

Ella's Kitchen, children's smoothies, launch, UK	C	A	B		A, C	B

Fortuna, juice, without added sugar campaign, PL	C	A				A

Bionade, organic soft drink, DE	C	A		A	C	B

ProViva, probiotic juice, SE	A				A	B

Ricola, cough drops "Who invented it?" DE/CH		A			A	

Unilever Flora pro-activ, margarine, "Test the nations heart", UK	A		A	A	B	

Kellogg's All Bran, cereals, "Feel great challenge", UK		A	A			A, C

Kellog's Special K, cereals, "Drop a jeans size", EU	B			B		A, B, C

Danone Activia, yoghurt, relaunch, UK	A, B			B	B	B

Nestlé Naturnes, baby food, launch, EU	B			B	B	

Coca Cola Kropla Beskidu, water, PL		B		C	A	

Coca Cola Aquarius, sports drink, "Humans are extraordinary", ES		A	A	A		

Kraft Kvikk Lunsj, chocolate, "Thanks for the tour", NO		A		C		

Note: only the cases mentioned as examples in the text are presented to ensure readability. A complete table of all cases can be provided on request

**Data and knowledge**:A = Nutritional evidence, B = Consumer insights, C = Trend awareness; **Emotions: **A = Emotional engagement, B = Simple naturalness; **Endorsement: **A = Vertical endorsement, B = Credible company; **Community: **A = Common values, B = Participation, C = Local relevance; **Media: **A = Matching media, B = Combined media, C = Media coverage; **Why and how: **A = Clear message, B = Close benefits, C = Achievability

#### "Data and knowledge"

The success of several cases appeared to be rooted in having some sort of superior data and knowledge. This knowledge can be scientific nutritional evidence or market and consumer behaviour insights, both forms of information that are actively sought by conducting or commissioning primary research. This knowledge can also be more intuitive knowledge and awareness of emerging trends that lead to "doing the right thing at the right time". Three cases provided examples of such knowledge. ProViva probiotic juice, successfully marketed by the south Swedish Skåne Dairy, was developed based on extensive nutritional research, and was consequently the first product officially granted a health claim in Sweden. Dairy giant Danone conducted thorough consumer research to prepare the relaunch of its Activia yoghurt. The relaunch was based on the new insight that most women complain about feeling "bloated" from time to time. In contrast, the producers of the innovative German-based organic soft drink Bionade had insufficient financial resources for such primary research, but appear to have recognised the macro-environmental trend toward organic and natural food and intuitively found the right approach to their young, urban and educated target market.

#### "Emotions"

Several examples derived their success from a communicational approach that, besides providing actual cognitive information, focused largely on the emotional side of the message, allowing for more emotional engagement by consumers. TV spots for the Swiss Ricola cough drops featured humour (the actor personifying the Swiss tries to catch other nations claiming to be the inventors), and the humorous spots for the Polish Fortuna juice without added sugar incorporated fear appeal ("bad" sugar cube characters try to seduce the fruits to mingle with them). A theme that appeared repeatedly was the emotional appeal to modern citizens' wish for "simplicity", "naturalness" and getting "back to nature", as can be seen in the spot for the Polish Kropla Beskidu water brand. In this spot, moments of happiness in everyday urban life are shown linked to water and enforced by an emotional song called "Touch of Joy".

#### "Endorsement"

Another characteristic observed was the use of different forms of endorsement to create trust and credibility. In a number of cases, endorsement is granted by involving, on a commercial basis, personalities that for various reasons are looked up to, such as respected or well-known celebrities, role models or heroes [45]. This KSF is called vertical endorsement, as opposed to peer-endorsement which operates on a horizontal level among people sharing the same status. One example is the actor William Shatner, who formerly played Captain Kirk, appearing in a series of humorous TV advertisements to encourage consumers to try Kellogg's All-Bran cereal as part of a "two-week challenge". The endorsement can also be an integral part of the company and its history, which adds credibility to the message. This was the case with the UK-based start-up company Ella's Kitchen: Ella is the daughter of the founder who was a father simply wanting to produce healthy and tasty smoothies for his children.

#### "Community"

One repeatedly observed aspect of successful commercial campaigns was summarised under the heading "community". This aspect was present in various forms. It could mean that the underlying message addresses or appeals to general human or social values and aims. It evokes a sense of "common ground" or shared values in consumers, creating the experience of being part of a community, linking consumers to common experience or tradition, and encouraging consumers to "appropriate" the campaign. For example, in the "anti-heroes" TV advertisements for Aquarius, a Spanish sports drink brand, humans are portrayed as being unpredictable and extraordinary, and this is depicted as a universal human characteristic. The spots tell stories of unexpected social heroes in different countries accomplishing the impossible. In combination with the song "Age of Aquarius", the message has reminders of ancestry and religious spirituality. Another example is the UK's "Test the nation's heart" campaign for Unilever's Flora Pro-activ margarine brand. During this campaign, a medical team tours the country performing blood pressure and cholesterol tests in front of supermarkets. The message of the campaign underlined the notion that it is the nation's problem and, therefore, a shared responsibility. A more direct use of the "community" cluster involves triggering active participation of consumers in the campaign or interaction between consumers and the campaign, often through the use of new media and social network sites. Nestlé's launch of the baby food brand Naturnes was accompanied by enabling interaction between mothers who had already tried the product via their website, and using Facebook to inform mothers about new developments. A third aspect of "community" relates to local origin or relevance, which unites consumers by linking the product to regional traditions or values. The water brand Kropla Beskidu, for example, links to the Polish mountain region of the Beskides, while the communication for Kraft Food's traditional Norwegian chocolate bar brand Kvikk Lunsj centres on Norwegian's widespread passion for Nordic outdoor activities.

#### "Media"

Choosing the best media match or the right combination of media for communication activities is crucial in commercial marketing, thus this type of success factor and related undertakings formed a separate cluster. TV was the main and sometimes only medium in a number of cases, as in the Ricola "Who invented it?" campaign. The use of TV is often justified by the enhanced and rich potential to appeal to emotions. In other examples, a good combination of media and often massive use of various media in a "360 degree" approach (reaching the consumer repeatedly from different angles) seemed to be important for success, as in the case of the relaunch of Danone's Activia. Examples from non-multinational companies indicate that media type and extensiveness of use might not exclusively be a matter of financial investment, and that communication can also be approached in alternative ways. In the case of Ella's Kitchen, the founder was able to negotiate a deal that allowed him free TV broadcast time in exchange for a share of later sales revenue. The Bionade campaign was, until recently, designed around sampling at events and word-of-mouth among the targeted trendsetters in Germany. Success in both campaigns was aided by favourable media attention and coverage, which led to positive publicity and thus non-funded mass media communication.

#### "Why and how"

The last cluster of KSF deals with conveying the marketing message, i.e. the facilitation of the consumer's ability to understand the main message and the motivation and ability to act accordingly. In the workshop, this cluster was summarised under the title "why and how". When explaining why consumers finally chose a product or change their consumption behaviour in favour of the advertised product, three aspects were common to the investigated cases. First, messages were simple and clear enough to be understood by the "average" consumer; second, motivation to act was encouraged by presenting long-term benefits, like improved health, combined with short-term benefits such as good taste, wellness or good looks; and, last, the way to achieve these benefits was explained step-by-step. One example of these three success factors is Kellogg's Special *K *cereal brand campaign. The use of the product was easily understood and benefits could be achieved in a foreseeable time period by following the formula "two bowls - two meals - two weeks". The "Drop a jeans-size" campaign focused on communicating well-being and the satisfaction of slimming as benefits, rather than purely emphasising the health outcome.

## Discussion

Public health campaigns for healthier eating differ from commercial food marketing campaigns in a number of aspects. This makes it challenging to transfer learning from one sector to the other [26]. The most important difference is the public sector's aim of improved public health versus the commercial success of an individual product, brand or company [46,47]. A major conceptual difference is that the focus in public campaigns is on general behaviours, rather than specific products, and they often call for avoidance instead of appealing to the desire for a particular object. Thus, the social marketing product is "inherently more complex than a commercial product" [12]. It is also far more complicated to measure a complex public health effect than a relatively straightforward effect on product sales. These limitations should be taken into account, and it should not be assumed that social marketing is the only or even the best way of promoting healthy eating [26]. However, it is still worthwhile considering whether, and in which ways, the key success factors of commercial food marketing can be adapted to the social marketing of, and public campaigns for, healthier food choices and eating habits. An improved and empirically-based understanding of commercial food marketing success factors is the first step in this direction.

To our knowledge, no other empirical study on the key success factors in food marketing, with a specific focus on the area of food and health, has been conducted to date. Some of the key success factors identified in this study have, however, been mentioned in previous articles on lessons to be learnt from food marketing, or have been highlighted in reviews or descriptions of examples of previous public policy activities for healthy eating. For example, a meta-analysis of public health campaigns concluded that the behaviours promoted should be "doable", the message "carefully crafted" and a variety of channels should be chosen to succeed [48]. A case study on public health campaigns and unfavourable industry practices focused on facilitators of success. It was found that success can be attributed to a range of factors, amongst others the local level of a campaign. Achieving media coverage or using a mix of channels was also found to be a factor for success [49]. Results of an expert workshop of national institutions in the USA stressed the importance of well-founded consumer insights and selecting the right media, combining media and achieving media coverage. Policymakers should appeal to and create emotional engagement, provide clear messages, and explain to citizens the steps they need to take to achieve benefits [47]. Another study on lessons to be learnt from food marketing also concluded that combined media and emotional engagement are crucial, and that focus should be placed, not just on health, but on the more short-term benefits [50]. In describing a famous public health branding case in the area of healthy eating, Evans and colleagues mention the need for a clear message that incorporates explanations of how to achieve results, and they emphasise the importance of a link to local relevance and enabling active participation. The cases that they highlight achieve credibility through the provision of a 'health brand' [23,51].

Comparison with previous studies shows that the clusters of success factors empirically attested by this study are in line with what has been reported previously. Several of the success factors, however, have not been mentioned previously, and we suggest that their prominence in our study can be attributed to the focus on food marketing related to nutrition and health. These factors suggest that the success of commercial food marketing: 1) depends on well-founded nutritional evidence; 2) can derive from being alert to trends; 3) might be based on appealing specifically to the modern citizens' wish for simplicity and naturalness; 4) is rooted in using vertical endorsement; and 5) depends on appealing to common values. We argue that health as a so-called "credence attribute" [52] might not only need attested scientific nutritional evidence, but also stronger enforcement through vertical endorsement, especially in an environment where there is an overwhelming amount of sometimes contradictory health information [47] that leads consumers to seeking guidance. Even though consumer interest in healthy food characteristics is not new anymore, its importance is ever-growing [53], and there are continuously new developments that require the market to be alert and react accordingly. The rising market share of organic foods [54] highlights that "naturalness" is a current trend. However, the desire for simplicity and naturalness might also be a reaction to information overload [55]. The repeatedly found appeal to "common values" might be of particular importance for health and food, due to the social and cultural importance of food [56], but also to the closely linked social implications for the individual's long-term health.

## Conclusions

The present study allows the conclusion that the key success factors identified under the six clusters are of importance for the success of commercial food marketing. The actual factors may not be new, especially to social marketers; however the major contribution of this study is pinpointing which factors are particularly crucial for success and, more specifically, which ones might be of greatest relevance for the area of nutrition and health. The present study also provides a model that can serve as a checklist for evaluating future public health nutrition campaigns, ex ante and ex post. Finally, the present study provides a large number of cases as examples, and these can serve as a source of ideas for future application, just as the case study approach suggests.

With regard to practical implications, the results of this study suggest that the public sector's food and health-related campaigns and interventions may benefit from adapting and applying the identified KSF that result in commercial success. This implies that public campaigns and interventions could be preceded by more intensive research on consumers' or citizens' behaviour and new societal trends. Public campaigns could also be strengthened by adding a stronger emotional appeal, especially by emphasising the desire for simplicity and naturalness, which is satisfied by healthy food and healthy eating. Promising approaches in public campaigns might be those that appeal to common values, and allow the experience of being part of a movement or group, or re-connecting to the local community. With regard to the choice of media, the use of social networks and emerging online social media, largely unexplored in public food and health communication in the EU so far, should also be considered. Healthy eating recommendations should also be simple, clear and achievable, and should stress short-term benefits alongside the long-term benefit of good health.

We agree with previous authors that public-private partnerships should be considered to a greater extent in the future to exchange expertise with regard to the success factors, but also to develop the agenda for the promotion of healthy eating even further [28,46,47,57]. Of course, possible pitfalls in this unequal relationship must be carefully assessed, and differences in culture as well as mutual scepticism will have to be overcome. Advertising, communication and consumer behaviour specialists might be able to play a role in bridging the gap between commercial and public applications of marketing techniques. The results of the present study imply that it might be beneficial for public-private-partnerships if private food companies increasingly share their consumer insights and trend awareness with public health policymakers, or co-finance activities in public health campaigns, such as celebrity endorsement. Further areas of cooperation, complementary to campaigns, should be joint efforts in shaping health-promoting environments for the consumers [19,47,57,58], and actions that counterbalance the market failures (as e.g. the information asymmetry, which contribute to obesity) [6,24]. In this regard, it must be noted that commercial success cases highlight the influence that nutrition and health claims can have on consumer food choice [59]. Thus, the study underlines the importance of scientifically founded claims; otherwise claims might be counterproductive for public health aims. Whether nutrition and health claims actually do improve public health does remain to be seen.

The next step in the EATWELL project is to focus on using the results of the present study to inform public policy. There is a need to put our finding into practice through a variety of policy measures, including the possibility of more public-private partnerships in the area of healthy eating.

## Competing interests

The authors declare that they have no competing interests.

## Authors' contributions

JAW designed the study with input from TBL and FPC. JAW conducted the secondary data collection and interviews. JAW, FPC and BN conducted the case analysis, wrote the case-descriptions and, with the help of TBL and WV, drafted the comparative analysis of the three subsets of cases. JAW, TBL and FPC conducted the comparative case analysis in the workshop. JAW wrote the draft manuscript and all authors contributed references, comments and revisions to the subsequent drafts. All authors made substantive intellectual contributions to the scientific content and approved the final manuscript.

## Pre-publication history

The pre-publication history for this paper can be accessed here:

http://www.biomedcentral.com/1471-2458/12/139/prepub
